# Supplementary material for: Characterizing and Targeting Genes Regulated by Transcription Factor MYBL2 in Lung Adenocarcinoma Cells
Source: Cancers (Basel). 2022 Oct 11;14(20):4979. doi: 10.3390/cancers14204979 (PMC9599349; doi:10.3390/cancers14204979)
Supplement: Supplementary file 1 [file cancers-14-04979-s001.zip › Supplementary Figures.pdf]

Supplementary information

Characterizing and targeting genes regulated by transcription factor MYBL2 in lung adenocarcinoma cells.

Yuri Lee, Zexun Wu, Seolyn Yang, Shannon M. Schreiner, Leonardo D. Gonzalez-Smith, and Suhn K. Rhie *

Department of Biochemistry and Molecular Medicine, Norris Comprehensive Cancer Center, Keck School of Medicine, University of Southern California, Los Angeles, California, 90033, United States of America

* Correspondence: rhie@usc.edu (S.K.R.); Tel.: +1-323-442-8145

Supplementary Figures

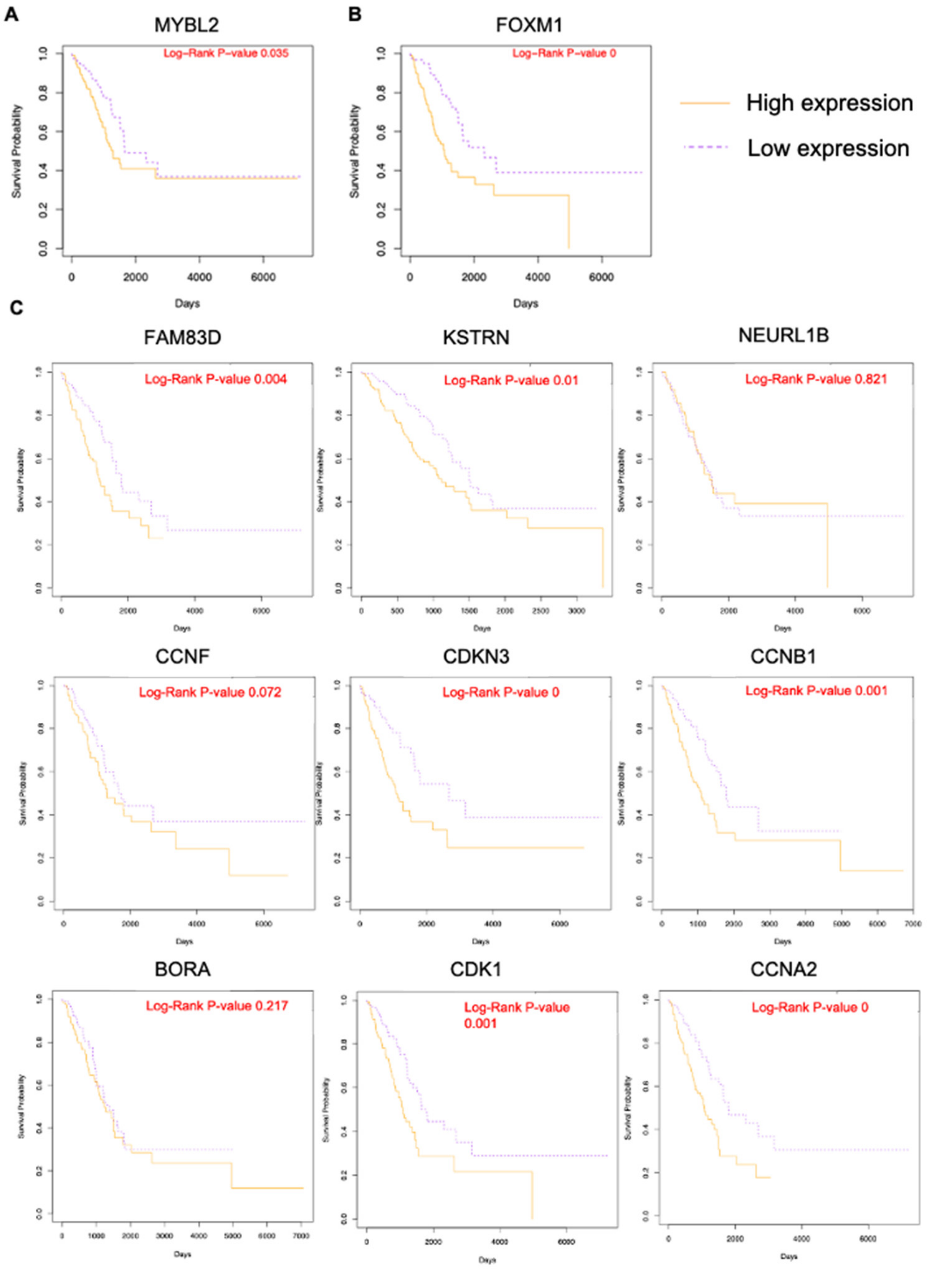

Supplementary Figure S1. Lung adenocarcinoma patient survival analysis. Kaplan-Meier survival plots comparing differences in survival between lung adenocarcinoma patient samples with the highest and lowest quartiles gene expression of (A) *MYBL2* (B) *FOXM1* and (C) target genes of MYBL2 and FOXM1. Survival analysis was conducted using TCGA LUAD gene expression and clinical data.

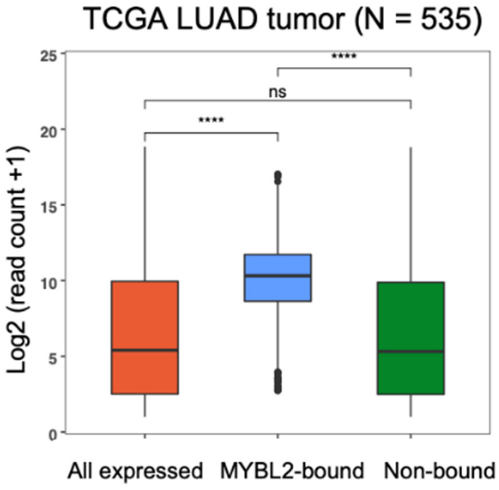

Supplementary Figure S2. MYBL2-bound genes are expressed high in lung adenocarcinoma. A boxplot showing gene expression levels of all expressed, genes with MYBL2-bound, and non-bound promoters. TCGA LUAD gene expression data and MYBL2 ChIP-seq in A549 lung adenocarcinoma cells were used to generate this plot (****, *P* value < 0.0001).

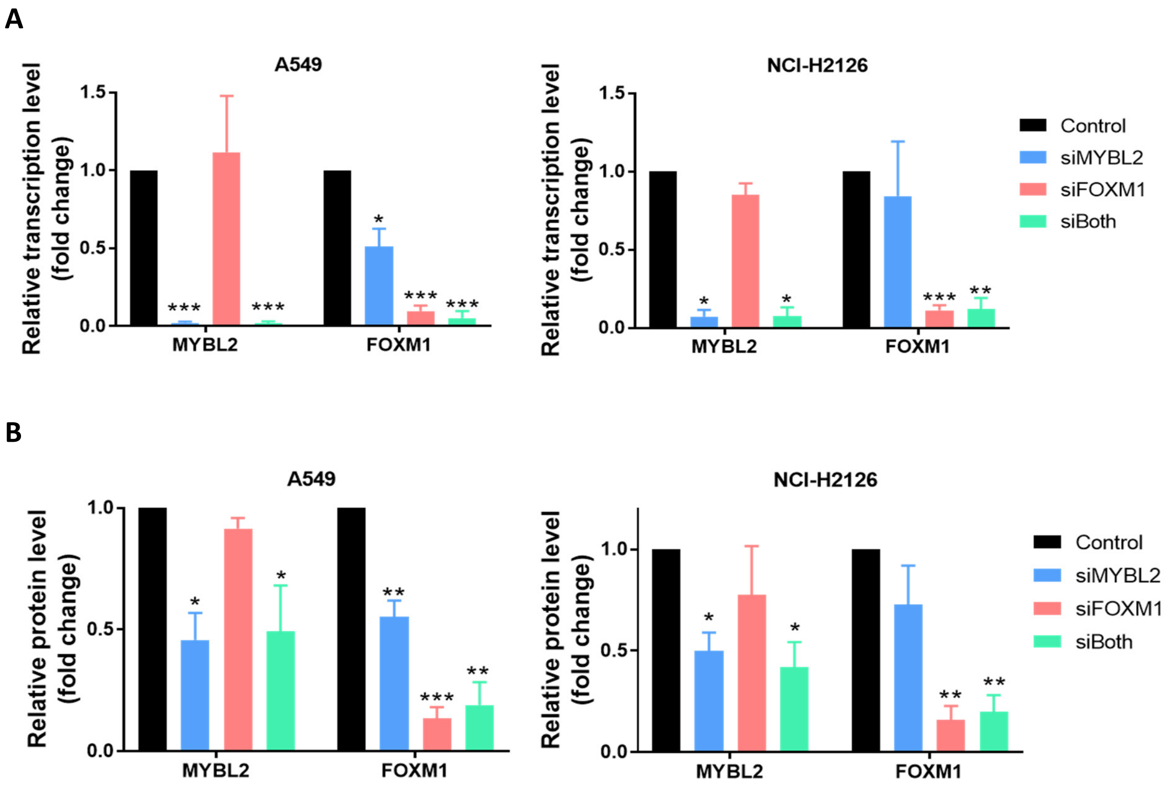

Supplementary Figure S3. The expression levels of MYBL2 and FOXM1 after knockdown experiments in A549 and NCI-H2126 lung adenocarcinoma cells. A549 and NCI-H2126 cells were transfected with negative control scrambled siRNA (NC), MYBL2 siRNA (siMYBL2), FOXM1 siRNA (siFOXM1) or both MYBL2 and FOXM1 (siBoth). (A) RT-qPCR and (B) Western blot experiments were performed to measure MYBL2 and FOXM1 gene and protein expression levels. Data represent the mean SE (n=3). *, *P* value ≤ 0.05, **, *P* value ≤ 0.01, ***, *P* value ≤ 0.001.

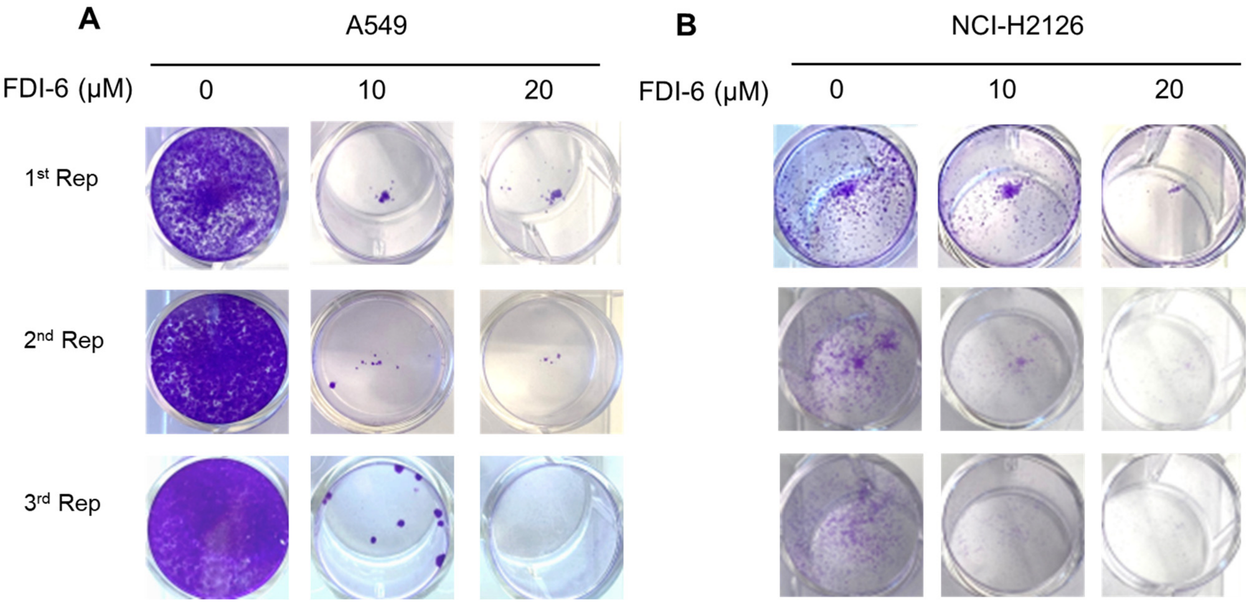

Supplementary Figure S4. Colony formation images of A549 and NCI-H2126 lung adenocarcinoma cells. (A) A549 cells were treated with different concentrations (0, 10, 20 μM) of FDI-6 for 24h, and then incubated for 14 days. (B) NCI-H2126 cells were treated with different concentrations (0, 10, 20 μM) of FDI-6 for 24h, and then incubated for 14 days (n=3).

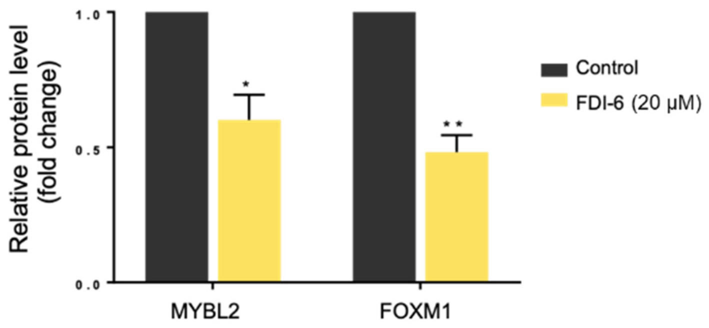

Supplementary Figure S5. The protein levels of MYBL2 and FOXM1 in the nucleus fraction after treatment of FDI-6 in A549 lung adenocarcinoma cells. A549 lung adenocarcinoma cells were treated with FDI-6 (20μM) or DMSO control. After 24h, western blot was performed by lysing nuclear fractions of A549 lung adenocarcinoma cells with 1X RIPA. Data represent the mean SE (n=3). *, *P* value ≤ 0.05, **, *P* value ≤ 0.01.

A Figure 4F Western blot analysis of FOXM1, MYBL2, CENPA and beta-actin (lane 4-5)

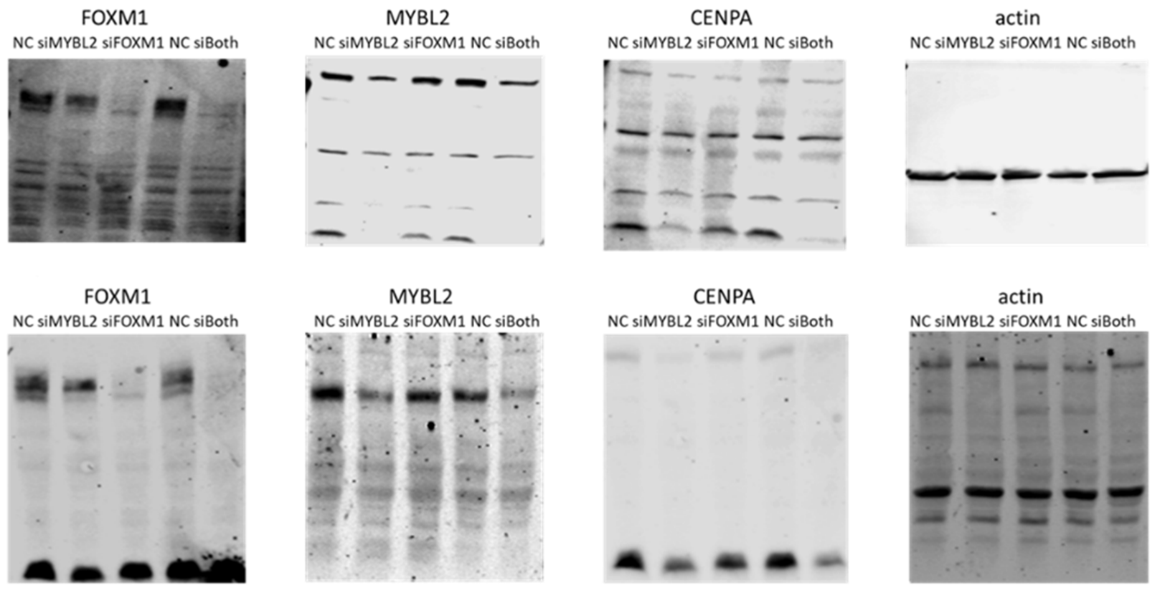

B Figure 5C Western blot analysis of FOXM1, MYBL2, and beta-actin (lane 3-4)

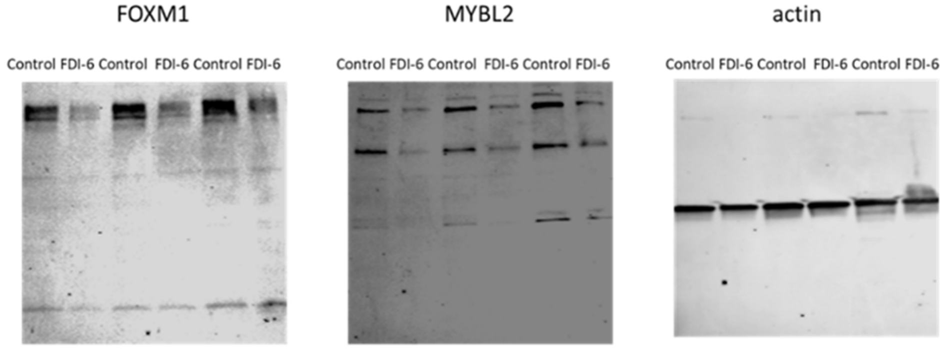

Supplementary Figure S6. Original western blot images. (A) Original western blot images of Figure 4F. Upper images are A549 cells, and lower image are NCI-H2126 cells. NC: negative control, siBoth: siMYBL2 and siFOXM1. (B) Original western blot images of Figure 5C.
